# Modulation of Metabotropic Glutamate Receptors as a Strategy to Improve the Efficacy and Safety of Ketamine as an Antidepressant

**DOI:** 10.3390/cells14241967

**Published:** 2025-12-11

**Authors:** Agnieszka Pałucha-Poniewiera

**Affiliations:** Department of Neurobiology, Maj Institute of Pharmacology, Polish Academy of Sciences, Smętna Street 12, 31-343 Krakow, Poland; nfpaluch@cyf-kr.edu.pl

**Keywords:** antidepressant, depression, ketamine, LY341495, M-5MPEP, mGlu_2_ receptor, mGlu_5_ receptor, RAAD, TRD

## Abstract

Since the introduction of the NMDA receptor antagonist (*S*)-ketamine for depression therapy, it has become evident that the glutamatergic hypothesis of depression, proposed over 20 years ago, was justified and based on solid foundations. A significant breakthrough with this drug is its ability to produce a rapid and relatively long-lasting antidepressant effect in patients who are resistant to traditional depression treatments, both pharmacological and non-pharmacological. However, alongside its beneficial effects, (*S*)-ketamine can cause several side effects that make it a less safe option. As a result, strategies are being explored to mitigate the risks associated with its use. These strategies include leveraging the shared mechanism of action between ketamine and various modulators of the glutamatergic system. Preclinical studies have shown that low doses of mGlu_2_ and mGlu_5_ receptor antagonists can enhance the therapeutic effects of ketamine or its enantiomers without producing the typical side effects associated with ketamine. This review discusses the research on this synergistic effect, the underlying mechanisms, and the role of mGlu_2_ and mGlu_5_ receptors in the antidepressant action of ketamine.

## 1. Introduction

(*S*)-ketamine is currently the only registered rapid-acting antidepressant drug (RAAD) that has proven effective in treating treatment-resistant depression (TRD) through both intravenous and intranasal administration [1,2,3,4]. However, the introduction of (*S*)-ketamine has not resolved ongoing research concerns regarding its mechanism of action, potential adverse effects, and its effectiveness in various mental health disorders [5,6]. (*S*)-ketamine is one of the two enantiomers of ketamine, which has been used in medicine for several decades as an anesthetic [7]. Ketamine is a non-selective drug that interacts with various molecular and cellular mechanisms, resulting in a wide range of effects, both beneficial and adverse. Notable adverse effects can include depersonalization, which is related to the dissociative properties of the substance, agitation, confusion and even psychotic effects. Additionally, (*S*)-ketamine carries a potential for abuse [6].

This raises several important questions: What specific mechanisms of action are responsible for ketamine’s antidepressant effects? Is it possible to separate the desired effects from the undesirable ones? Can we minimize adverse effects while maintaining therapeutic effectiveness? How close are we to finding an alternative RAAD that is equally effective but has a lower potential for abuse? To address these questions, further exploration into the mechanisms of action of ketamine is necessary, utilizing both animal models and human studies.

A comprehensive discussion of the various mechanisms of action of ketamine is beyond the scope of this review. However, there are several important points to highlight. First, the antidepressant effect of ketamine follows a U-shaped curve, indicating that its effects are not observed at either low doses or high (anesthetic) doses [8]. Second, ketamine’s direct mechanism of action is primarily related to its modulation of glutamatergic transmission, specifically targeting the ionotropic glutamate receptor, NMDA [7]. Ketamine acts as a channel blocker at the phencyclidine (PCP) binding site of this receptor, suggesting that its action may be inhibitory. Interestingly, this inhibition may have the opposite effect; blocking NMDA channels on GABAergic interneurons in the prefrontal cortex (PFC) results in the disinhibition of glutamatergic pyramidal cells, leading to increased neuronal activity [9]. This heightened activity of pyramidal neurons in the PFC, which occurs following subanesthetic doses of ketamine, appears to initiate a series of neuroplastic changes in this area [10,11]. These changes may be modulated by factors that regulate the translation process necessary for producing new proteins, which are essential for forming new synaptic connections. mTOR kinase has been proposed as a key regulator of protein translation in this context [12]. Another proposed factor regulating the translation process, which plays a crucial role in ketamine’s antidepressant action, is eEF2 kinase. Research demonstrated its key role in the hippocampus, forming the basis for the theory of rapid homeostatic plasticity, highlighting the specific function of BDNF/TrkB signaling [13].

Interestingly, there are strong indications that ketamine’s mechanism of action is independent of direct NMDA receptor blockade [14,15]. This principle also applies to the antidepressant-active ketamine metabolite, (*2R*,*6R*)-HNK [15]. This finding offers hope for achieving therapeutic effects without the adverse effects commonly associated with NMDA receptor blockade, particularly since other NMDA receptor channel blockers do not replicate the ketamine-like antidepressant effects in humans [16].

On the other hand, activation of NMDA receptors, particularly through the GluN2A subunit, appears to be necessary for inducing the rapid antidepressant effects of not only ketamine but also other potential RAADs [8]. Thus, enhancing NMDAR signaling to promote NMDAR-dependent long-term potentiation (LTP)-like synaptic potentiation has been proposed as an effective antidepressant strategy [8]. Furthermore, studies indicate that there are additional targets, unrelated to glutamatergic receptor regulation, that also contribute to ketamine’s antidepressant response. These targets primarily include opioid receptors, sigma receptors, the TrkB receptor, and others [17,18].

While (*S*)-ketamine is the only RAAD registered for the treatment of TRD, there is also significant therapeutic potential in its second enantiomer, (*R*)-ketamine, as well as in (*RS*)-ketamine and some of its metabolites, including (*2R*,*6R*)-HNK [15,19]. Additionally, substances that modulate glutamatergic transmission through mGlu receptors show promise as RAADs; however, current evidence is primarily based on animal studies. These substances primarily include functional antagonists of the mGlu_5_ and mGlu_2/3_ receptors [20,21,22].

Notably, many cellular and molecular mechanisms responsible for the rapid antidepressant effects of ketamine have also been observed with mGlu_5_ and mGlu_2/3_ antagonists. This suggests a convergence in the mechanisms of action for these substances, despite their different receptor targets. Key mechanisms include: activation of mTOR, which is crucial for enhancing neuroplasticity and contributing to the antidepressant effect [23,24,25,26], the central role of the AMPA receptor [27,28,29,30], downstream activation of the NMDA receptor [8] (Zanos et al., 2023), and dependence on BDNF/TrkB signaling in their action [26,31]. Furthermore, shared mechanisms involving serotonergic and dopaminergic neurotransmission have been proposed as contributing to the antidepressant actions of both ketamine and mGlu_2/3_ receptor antagonists [24,27,28,30,32]. To our knowledge, the only significant mechanism of rapid ketamine’s antidepressant action not demonstrated for mGlu_2/3_ antagonists is its dependence on the regulation of eEF2 kinase [13].

In addition, mGlu_2/3_ and mGlu_5_ receptor antagonists have been shown to replicate the behavioral effects of ketamine in various tests and animal models of depression. Notably, these antagonists exhibited effects that lasted 24 h or longer after a single or short-term administration. This prolonged activity is characteristic of RAADs and differs from traditional antidepressants, such as selective serotonin reuptake inhibitors (SSRIs) [21,25,29,30,31,33]. Moreover, mGlu_2/3_ receptor antagonists (like LY341495, MGS0039, and TP0178894) and mGlu_5_ receptor negative allosteric modulators (NAMs), such as M-5MPEP, demonstrated antidepressant effects in animal models of depression, including the chronic unpredictable mild stress (CUMS) and social defeat stress (SDS). These compounds induced a rapid reversal of modeled depression symptoms following a single or short-term administration, a trait associated with RAADs, but not with classical antidepressants [34,35,36,37,38]. Importantly, from a practical standpoint, mGlu_2/3_ antagonists have shown a favorable safety profile, as they do not produce neurotoxic, motor, cognitive, or abuse-related effects similar to those of ketamine [39].

Given the significant overlap in the mechanisms of action between ketamine and mGlu_2/3_ and mGlu_5_ receptor antagonists observed in animal models, an important question arises: could these compounds act synergistically? If so, could a subtherapeutic dose of ketamine be enhanced by the co-administration of an mGlu_2_ antagonist or an mGlu_5_ antagonist? This approach may help reduce the adverse effects associated with ketamine. Recent studies have provided several insights on this topic. They explore not only the potential to enhance the antidepressant effects of ketamine through mGlu_2/3_ and mGlu_5_ antagonists but also the effects of its enantiomers, (*S*)-ketamine and (*R*)-ketamine. Additionally, research indicates that mGlu_2_ and mGlu_5_ receptors play a role in the mechanisms through which ketamine, its enantiomers, and its active metabolites exert their effects.

## 2. The Involvement of the mGlu2 Receptor in the Effects of Ketamine

The initial data regarding the functional relationship between mGlu_2/3_ receptor activation and ketamine’s effects in the brain were presented in studies by Lorrain et al. [40,41]. These studies demonstrated that mGlu_2/3_ receptor agonists could reverse certain behavioral effects induced by ketamine, such as hyperlocomotion, and block the release of glutamate, norepinephrine, and dopamine triggered by ketamine, particularly in the medial prefrontal cortex (mPFC). This suggests that mGlu_2/3_ receptor agonists may have potential for treating symptoms of psychosis through a mechanism that reduces ketamine (or PCP)-induced glutamatergic neurotransmission in the mPFC. Notably, these studies indicated that the relationship between ketamine and mGlu_2/3_ agonists is mainly one of negative feedback. The mechanism underlying this effect is likely related to the synaptic distribution and functional characteristics of the mGlu_2/3_ receptor. mGlu_2_ receptors are primarily located presynaptically in the preterminal regions of axons and function as inhibitory autoreceptors during periods of high excitation. They are abundantly expressed in limbic areas, including the cerebral cortex, hippocampus, and amygdala [42,43,44]. Consequently, mGlu_2_ receptor agonists inhibit glutamate release, which may result in reduced ketamine-enhanced glutamate levels, thereby decreasing ketamine-induced neurochemical and behavioral effects. Subsequent studies have confirmed this functional relationship between mGlu_2_ receptor activation through agonists or positive allosteric modulators (PAM) and ketamine. For instance, it was found that pretreatment with the mGlu_2/3_ receptor agonist LY379268 significantly reduced ketamine-induced blood oxygenation level-dependent (BOLD) signals in the rat brain [45]. Similar findings were confirmed in humans, where mGlu_2/3_ receptor agonists (LY2140023 and LY2979165) also reduced the ketamine-evoked BOLD phMRI signal [46]. Additionally, another indicator of neuronal activation, the cortical quantitative EEG (qEEG) gamma power evoked by ketamine, was found to decrease following pretreatment with the mGlu_2/3_ agonist LY379268 [47,48,49] or PAM TASP0433864 [50].

A stronger relationship has been observed between the activity of mGlu_2_ receptor ligands and ketamine concerning its antidepressant effects. The initial evidence for this association was provided by a comparative study conducted by Witkin et al. [30], which highlighted several physiological, pharmacological, and behavioral similarities between ketamine and the mGlu_2_ receptor antagonist LY3020371. Notably, the study demonstrated that in the forced swim test (FST) in rats, the mGlu_2/3_ receptor agonist LY354740 completely blocked the antidepressant-like effects of ketamine [30]. Further investigation by Zanos and colleagues confirmed the mGlu_2/3_ receptor-dependent activity of the antidepressant doses of ketamine in mice. They utilized a hyperthermia assay induced by the mGlu_2/3_ receptor agonist LY379268 for this purpose [51]. This effect was replicated by (*2R*,*6R*)-HNK, a ketamine metabolite with antidepressant properties, which functions through a mechanism that does not depend on NMDA receptor inhibition. Conversely, NMDA receptor antagonists, which do not produce antidepressant effects—such as channel blockers at the PCP-binding site like MK-801 or Ro 25-6981, a GluN2B-specific NMDA receptor antagonist—did not induce the same effects [51]. These data suggest that the mechanism preventing LY379268-induced hyperthermia by ketamine is unlikely to involve NMDA receptor inhibition. Additionally, experiments using mice that genetically lack the Grm3 or Grm2 gene revealed that the effect is associated with the mGlu_2_ receptor but not the mGlu_3_ receptor [51]. Therefore, it has been established that the antidepressant action of ketamine and its active metabolite relies on the activation of the mGlu_2_ receptor, rather than directly blocking the NMDA receptor. Furthermore, an enhancement of high-frequency (gamma) qEEG oscillations—an in vivo marker of neuronal excitation—was observed following the administration of LY341495 and (*2R*,*6R*)-HNK [51]. These findings imply that both mGlu_2_ receptor antagonists and (*2R*,*6R*)-HNK act in a manner dependent on the mGlu_2_ receptor to enhance high-frequency neuronal activity, which may be related to their antidepressant effects. Another study indicated that (*2R*,*6R*)-HNK does not directly bind to the mGlu_2_ receptor; therefore, the relationship between ketamine and the mGlu_2_ receptor appears to involve an indirect mechanism [52].

## 3. Synergistic Effects of Ketamine and mGlu_2/3_ Receptor Antagonists

Due to the high similarity in the mechanisms of rapid antidepressant action between ketamine and the mGlu_2/3_ antagonist, Podkowa et al. [53] proposed, for the first time, combining subthreshold doses of both compounds. This approach aimed to investigate their potential synergistic effects while minimizing the side effects commonly associated with ketamine. In their study using the FST in rats, the authors confirmed that the mGlu_2/3_ antagonist LY341495 enhanced the effects of a subthreshold dose of ketamine both 40 min and 24 h after administration. The results provided the first evidence that combining these subthreshold doses of ketamine and LY341495 produces synergistic antidepressant-like effects without inducing the typical adverse effects associated with higher, antidepressant doses of ketamine [53]. Similarly, Zanos et al. [51] conducted independent research in mice, examining how the physiological actions of ketamine or its active metabolite (*2R*,*6R*)-HNK relate to mGlu_2_ receptor signaling. They aimed to determine whether these effects could be reflected in animal models predictive of antidepressant activity. To investigate the synergistic effects of mGlu_2/3_ receptor antagonists when combined with ketamine, subeffective doses of LY341495 and ketamine were co-administered and assessed using the FST in mice. The study found that this drug combination, as well as the combination of LY341495 and (*2R*,*6R*)-HNK, produced significant antidepressant-like effects both 1 h and 24 h after administration [51]. Furthermore, the subeffective doses of LY341495 and (*2R*,*6R*)-HNK resulted in a synergistic enhancement of cortical qEEG gamma power, a marker of target engagement associated with antidepressant efficacy [51].

In line with the hypothesis that a convergent mechanism of action exists between mGlu_2/3_ receptor inhibition and the effects of ketamine, subsequent studies were conducted using a model of depression based on unpredictable chronic mild stress (CUMS) in mice. The study demonstrated a synergistic effect when ketamine and LY341495 were administered together in subthreshold doses over three consecutive days. This combination effectively eliminated the stress-induced effects of apathy (measured by self-grooming time), anhedonia (assessed through sucrose consumption preference), and helplessness (evaluated in the tail suspension test, TST) [38]. Importantly, the coadministration of low doses of ketamine and LY341495 did not cause the hyperactivity typically associated with NMDA channel blockers. Furthermore, it did not impair short-term memory in the novel object recognition (NOR) test or disrupt motor coordination in the rotarod test, indicating a favorable safety profile for this treatment [38].

A recent study by Rafało-Ulińska et al. [54] has provided new insights into the effects of the combined administration of an mGlu_2/3_ antagonist along with each of the ketamine enantiomers, (*R*)-ketamine and (*S*)-ketamine, analyzed separately. The study’s findings indicated that in the TST, the effects of both ketamine enantiomers were enhanced when administered with LY341495, an mGlu_2/3_ antagonist, 60 min after administration. These enhanced effects were found to depend on the activation of the AMPA receptor, rather than the TrkB receptor. Furthermore, in the CUMS model of depression, which enables to assess the effects of fast-acting antidepressants, it was observed that 24 h after treatment, only (*R*)-ketamine showed a significantly enhanced effect when combined with the mGlu_2/3_ receptor antagonist. This enhancement was evident across all tests measuring anhedonia, apathy, and helplessness. In contrast, no enhancement was seen when (*S*)-ketamine was combined with LY341495 in any of the tests used [54]. This suggests that (*R*)-ketamine is primarily responsible for the enhanced antidepressant effects observed with the racemic mixture of ketamine, which contains equal parts of both enantiomers. Notably, the antidepressant effects of the combination of (*R*)-ketamine and LY341495 persisted for 3 to 4 days following administration [54]. The significance of this study lies in its demonstration of the differing mechanisms through which ketamine enantiomers act in relation to the mGlu_2_ receptor. Additionally, it highlights the potential for safe and effective therapy; (*R*)-ketamine tends to produce fewer adverse effects, and the co-administration of an mGlu_2_ antagonist may allow for a further reduction in the dosage of (*R*)-ketamine required.

## 4. Possible Mechanism for the Enhancement of Ketamine’s Antidepressant Effects Through Antagonism of the mGlu_2_ Receptor

The question arises: What mechanisms enhance the antidepressant effects of ketamine (or (*R*)-ketamine) when combined with the mGlu_2_ receptor antagonist, and which brain areas might be involved in these processes? The first study showing that the mGlu_2_ receptor antagonist potentiates the effects of ketamine in the FST in rats suggested a possible mechanism for this interaction [53]. It was found that this drug combination caused a rapid increase (40 min post-administration) in mTOR phosphorylation in both the hippocampus and the prefrontal cortex (PFC). This suggests that mTOR kinase-dependent protein translation may play a role in this enhancement. However, only in the hippocampus was there a significant increase in the PSD-95 protein level, comparable to the antidepressant dose of ketamine, 24 h after administering the tested compounds. PSD-95 is a crucial scaffolding protein located at the postsynaptic density of excitatory synapses, essential for synaptic transmission and plasticity [55]. It has also been implicated in the rapid antidepressant effects of ketamine [12]. Therefore, it seems likely that increased neuroplasticity in the hippocampus contributes to the antidepressant-like activity of the combination of ketamine and LY341495 [53]. A subsequent behavioral study by the same research group demonstrated that an AMPA receptor antagonist completely blocked the antidepressant-like effects of ketamine administered alongside LY341495 in the FST in rats. Additionally, a TrkB receptor antagonist, ANA-12, inhibited this effect at one of three studied time points (3 h post-administration) [56]. In a depression model using chronic unpredictable mild stress (CUMS), the prolonged antidepressant-like effect of LY341495 co-administered with (*R*)-ketamine was found to rely on the activation of either AMPA or TrkB receptors. This treatment also restored the stress-reduced BDNF levels in the hippocampus [54]. Collectively, these findings indicate that the induction of neuroplastic processes involving AMPA/mTOR/BDNF/TrkB signaling is likely a key mechanism behind the synergistic antidepressant effect of the mGlu_2_ receptor antagonist in conjunction with ketamine and its active metabolites or enantiomers. This conclusion is further supported by studies showing that the neuroplastic effects of LY341495, including dendritic outgrowth and increased spine density in rat hippocampal neurons, required activation of AMPA receptor-mTOR signaling. Additionally, LY341495-induced BDNF expression was inhibited by pretreatment with AMPA and mTORC1 antagonists (NBQX and rapamycin, respectively) [57].

Studies by Zanos and colleagues on mice have significantly advanced our understanding of the synergistic effects of ketamine and the mGlu_2_ antagonist [51]. The authors demonstrated that combining subeffective doses of LY341495 and (*2R*,*6R*)-HNK resulted in increased gamma qEEG oscillations, which are markers of neuronal excitation in vivo [51]. Recent studies have indicated that the antidepressant action of (*2R*,*6R*)-HNK in the CA1 region of the hippocampus is associated with the regulation of presynaptic rapid potentiation. This process stimulates priming mechanisms that promote the persistent formation of NMDA-activation-dependent LTP and enhance synaptic plasticity [58]. Of note, the induction of synaptic potentiation by (*2R*,*6R*)-HNK does not require NMDA receptor activity; however, NMDA receptor activity is essential for maintaining synaptic priming [58]. Thus, it is possible that presynaptically located mGlu_2_ receptors, which are significant in the antidepressant action of ketamine in an NMDA-independent manner, may also play a role in the induction of (*2R*,*6R*)-HNK-induced synaptic potentiation, that also occurs presynaptically and is independent of NMDA receptor activity.

**Figure 1:**
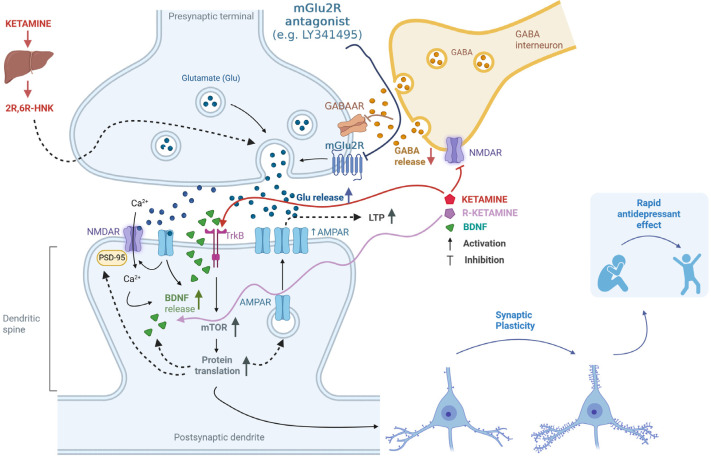
Putative mechanisms for the enhancement of ketamine’s antidepressant effects through antagonism of the mGlu_2_ receptor. Ketamine works by inhibiting NMDA receptors located on GABAergic interneurons [9]. This inhibition reduces GABA release, leading to the disinhibition of glutamatergic neurons [9]. Additionally, mGlu_2_ receptors (mGlu_2_R) are located on glutamatergic neurons and act as autoreceptors [42,43,44]. When mGlu_2_R antagonists (e.g., LY341495) block these receptors, it results in increased glutamate release and a consequent enhancement of the ketamine effect [30,38,51,53]. One of ketamine’s metabolites, (*2R*,*6R*)-HNK, also plays a role in regulating glutamate release through a presynaptic mechanism, further intensifying its effects [15,33,58]. The released glutamate stimulates AMPA receptors, and upon membrane depolarization, NMDA receptors are activated. This activation allows calcium ions to enter the cell, initiating intracellular signaling cascades and promoting the release of BDNF. BDNF then activates TrkB receptors and initiates various intracellular pathways, including the activation of the mTOR kinase pathway [12,53,54]. This leads to increased protein translation and the production of essential proteins for forming new synapses, such as AMPA receptor subunits and PSD-95 protein, as well as BDNF [10,11,12,13,53]. Additionally, ketamine can directly bind to the TrkB receptor [17]. These processes enhance synaptic plasticity, which is fundamental to the rapid antidepressant effect [10,11,12,13,54,59]. Created in BioRender. Pałucha-Poniewiera, A. (2025) https://BioRender.com/1owyasp (accessed on 17 November 2025).

Additionally, using electrophysiological techniques, it was found that co-administering LY341495 with (*R*)-ketamine prevented changes in glutamatergic transmission and synaptic plasticity induced by CUMS in the mouse PFC. Specifically, the amplitude of field potentials, LTP, and paired-pulse responses—indicators of short-term synaptic plasticity—were affected [59]. There is a proposal to enhance NMDA receptor-dependent LTP-like synaptic potentiation as an effective antidepressant strategy [8]. The synergistic action of LY341495 and (*R*)-ketamine aligns with this strategy, especially since previous research has shown that blocking mGlu_2/3_ receptors increases LTP [60]. The proposed mechanisms of action of the mGlu_2_ antagonist in combination with ketamine or its enantiomers or an active metabolite are comprehensively shown in Figure 1.

## 5. The Involvement of the mGlu_5_ Receptor in the Antidepressant Action of Ketamine

The mGlu_5_ receptor plays an important role in the mechanism by which ketamine exerts its antidepressant effects. Human studies support this idea, as they demonstrate a decrease in mGlu_5_ receptor availability following intravenous administration of ketamine. For example, a significant reduction in the binding of the selective radioligand [11C]ABP688 to the mGlu_5_ receptor was observed in several regions of the human brain, including the medial prefrontal cortex, amygdala, and hippocampus, after ketamine infusion. This finding was made using high-affinity positron emission tomography (PET) [61]. Another PET study confirmed the decrease in mGlu_5_ receptor availability in patients receiving intravenous ketamine. This effect lasted for up to 24 h after administration and was correlated with ketamine’s antidepressant effects [62]. In animal studies using rodent models of depression, such as prenatal stress in rats and CUMS in mice, it was found that stress-induced overexpression of the mGlu_5_ receptor was reversed by an antidepressant dose of ketamine [63] or (*R*)-ketamine [64]. Additionally, knocking down mGlu_5_ receptors led to decreased depression-like behaviors [63], indicating the receptor’s involvement in the therapeutic effects of RAADs. The hippocampus has also been identified as a potential brain region involved in these effects [63,64]. Possible mechanisms explaining the reduction in mGlu_5_ receptor availability following ketamine administration, or its enantiomers, include either the internalization of the mGlu_5_ receptor induced by ketamine or conformational modifications within the mGlu_5_ receptor that affect its expression on the cell surface. These mechanisms will be analyzed in detail below [61,65].

Based on these data, it can be concluded that the mechanism of action of ketamine, or its enantiomers, may involve a reduction in mGlu_5_ receptor activity. This finding aligns with well-documented evidence regarding the potential antidepressant effects of mGlu_5_ receptor antagonists and NAMs [20,66,67,68]. Supporting this hypothesis, studies using behavioral methods have demonstrated the mGlu_5_ receptor-dependent mechanism of ketamine’s action. For example, in the FST in rats, the activation of the mGlu_5_ receptor through the positive allosteric modulator CDPPB was found to inhibit the antidepressant-like effects of ketamine [69]. Conversely, the beneficial effects of ketamine appear to require reduced mGlu_5_ receptor activity [69]. Thus, the relationship between NMDA and mGlu_5_ receptors seems to be one of negative feedback. This type of interaction has been observed in other in vivo studies. For instance, research on the anesthetic properties of ketamine has shown that mGlu_5_ receptor agonists, such as DHPG and CHPG, reduce ketamine-induced anesthesia in mice. In contrast, an mGlu_5_ receptor antagonist, MPEP, enhances this effect [70]. Similarly, studies investigating the psychotic-like properties of ketamine in mice indicated that ketamine-induced schizophrenia-like behaviors could be reversed by mGlu_5_ receptor agonists [71].

## 6. Synergistic Effects of Ketamine and mGlu_5_ Receptor Antagonists and NAMs

As stated above, research indicates that reducing mGlu_5_ receptor activity is essential for the antidepressant effects of ketamine and its enantiomers. Consequently, a natural area of investigation has been to determine whether simultaneous blocking of the mGlu_5_ receptor—using selective antagonists or negative allosteric modulators (NAMs)—could enhance the antidepressant effects of ketamine. Several studies utilizing screening tests and animal models of depression have shown that administering mGlu_5_ receptor NAMs at subthreshold doses can actually amplify the antidepressant effects of ketamine. For example, combining the mGlu_5_ receptor NAM MTEP with ketamine resulted in significant synergistic antidepressant effects in the FST in rats [69]. Similar findings were reported in the TST in mice, where each enantiomer of ketamine was individually analyzed. The study revealed that the partial NAM of the mGlu_5_ receptor, M-5MPEP, administered at a subthreshold dose, enhanced the antidepressant effect of (*R*)-ketamine but not (*S*)-ketamine [21]. This specific effect was further confirmed using the CUMS model of depression in mice. The combination of subthreshold doses of (*R*)-ketamine and M-5MPEP effectively reduced symptoms of apathy and anhedonia induced by CUMS. This synergistic effect was related to changes in the levels of eEF2 and TrkB but not mTOR, in the hippocampus [64].

The significance of these recent studies lies not only in identifying (*R*)-ketamine as the enantiomer specifically associated with the potentiating effects of mGlu_5_ NAMs but also in utilizing a partial mGlu_5_ receptor NAM, M-5MPEP [72]. This partial NAM appears to be both effective and safe compared to previously used full mGlu_5_ NAMs like MPEP and MTEP. While full mGlu_5_ receptor NAMs have demonstrated significant antidepressant potential in animal studies, their progression to subsequent research stages has been impeded. This is primarily due to severe side effects, such as psychotomimetic effects and cognitive dysfunction [73,74]. Only studies on partial mGlu_5_ receptor NAMs, which induce less than 100% of the effects achieved by full NAMs at maximum concentrations, have raised hopes for their therapeutic applications. These compounds have demonstrated desirable effects, including anxiolytic, antidepressant, and anticocaine abuse properties, at doses that do not cause psychotomimetic side effects [72,75]. The broader therapeutic window of partial mGlu_5_ NAMs, combined with the similarity of some of their antidepressant mechanisms to RAADs, suggests that these compounds could improve the safety and efficacy of RAADs.

In summary, augmenting subthreshold doses of (*R*)-ketamine through the co-administration of a partial mGlu_5_ receptor NAM may offer an effective and safe treatment option for depression. However, this topic warrants further investigation, particularly in depressed patients. Studies demonstrating the antidepressant-like effects of administering subthreshold doses of an antagonist or NAM of mGlu_2_ or mGlu_5_ receptors alongside subthreshold doses of ketamine, its enantiomers, or a metabolite are summarized in Table 1.

## 7. Putative Mechanism of Potentiation of Ketamine’s Antidepressant Effect by mGlu_5_ Receptor Antagonism

What mechanism accounts for the strong functional association between ketamine and mGlu_5_ receptor ligands? To answer this question, it may help to consider some relevant and well-documented data. First, mGlu_5_ receptors interact with NMDA receptors through molecular mechanisms that arise from their physical and functional connection. Electrophysiological, biochemical, and molecular studies have demonstrated that the activation of the NMDA receptor enhances the function of the mGlu_5_ receptor. Conversely, activation of the mGlu_5_ receptor leads to an increase in NMDA receptor currents [76,77,78,79,80]. This functional interdependence is the result of an indirect physical association between mGlu_5_ receptors and NMDA receptors, which is mediated by Shank and Homer proteins. The Shank protein, part of the NMDA receptor-associated PSD-95 complex, binds to scaffolding Homer proteins, which are directly connected to the C-terminal tail of the mGlu_5_ receptor [81,82].

Notably, Homer proteins play a crucial role in regulating the interaction between mGlu_5_ and NMDA receptors. The long form of Homer1, known as Homer1b, facilitates a stable functional connection between the mGlu_5_ receptor and the NMDA receptor via the Shank protein. In contrast, the short form, Homer1a, disrupts this interaction [83,84]. Homer1a competes with Homer1b for binding to the mGlu_5_ receptor, which results in the uncoupling of mGlu_5_ from the Shank proteins that link it to the NMDA receptor [85]. Interestingly, ketamine can interfere with the regulatory mechanisms of Homer proteins, thereby affecting the mGlu_5_-NMDA receptor interaction. For example, ketamine has been shown to induce the expression of Homer1a in the cortical regions of the rat brain while simultaneously reducing the expression of Homer1b and PSD-95 in both cortical and striatal regions [86]. Furthermore, the antidepressant effects of ketamine are diminished when Homer1a is knocked down using siRNA in the medial prefrontal cortex (mPFC) [87]. This suggests that ketamine increases the ratio of Homer1a to Homer1b, leading to the disassembly of the indirect connection between mGlu_5_ and NMDA receptors involving Shank and Homer proteins [88]. On the other hand, disconnection of the mGlu_5_ receptor from Homer1b has been shown to increase the lateral mobility of the mGlu_5_ receptor and promote its direct interaction and co-clustering with the NMDA receptor in hippocampal neurons [89].

Recently, Elmeseiny and Müller [90] proposed an intriguing and comprehensive mechanism for ketamine’s antidepressant action, linking NMDA inhibition with mGlu_5_ receptor modulation. This concept is based on the important role of Homer proteins in regulating the functional connection between mGlu_5_ and NMDA receptors. The authors suggest that ketamine uncouples the mGlu_5_ receptor from the Shank protein complex by inducing Homer1a. With the increased mobility of the mGlu_5_ receptor and its lateral diffusion into the synapse, it can directly associate with the NMDA receptor. According to the authors, this complex could integrate molecular information and facilitate the prolonged antidepressant effects observed even after the drug has been cleared from the brain [90].

**Figure 2:**
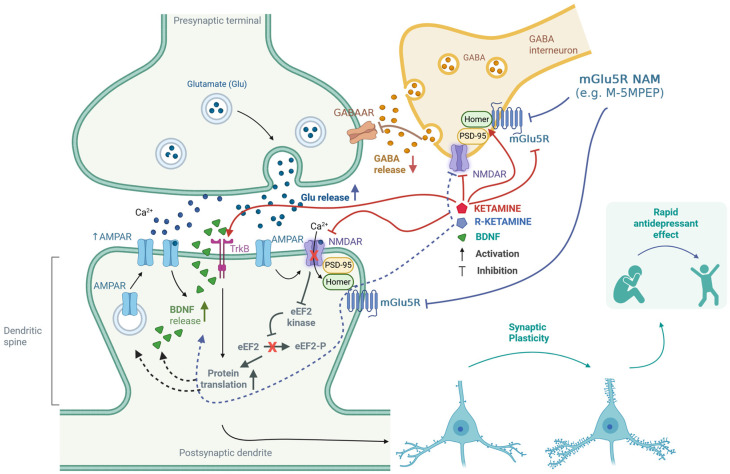
Putative mechanisms for the enhancement of ketamine’s antidepressant effects through antagonism of the mGlu_5_ receptor. Ketamine inhibits GABAergic interneurons by blocking NMDA receptors, which leads to the disinhibition of glutamatergic neurons [9]. Furthermore, both ketamine and (*R*)-ketamine reduce the availability of mGlu_5_ receptors [61,62,63,64]. These receptors are functionally linked to NMDA receptors through the Homer proteins and Shank proteins—a part of the PSD-95 complex [81,82]. Homer proteins play a crucial role in regulating the interaction between NMDA and mGlu_5_ receptors [83,84,85]. Ketamine can disrupt the regulatory mechanisms of Homer proteins, affecting the interplay between mGlu_5_ and NMDA receptors [86,87,88,89]. The release of glutamate stimulates AMPA receptors, which subsequently activates NMDA receptors. This process triggers the release of BDNF, further activating TrkB receptors [10,13]. Additionally, ketamine can directly bind to the TrkB receptor [17]. The activation of TrkB initiates intracellular signaling cascades that promote protein translation. The regulation of eEF2 kinase [13] is essential in this context—the synergistic effect of (*R*)-ketamine and the mGlu_5_ receptor NAM was linked to changes in the levels of eEF2 and TrkB, but not mTOR [64]. These processes enhance synaptic plasticity, which is fundamental to the rapid antidepressant effect [10,11,12,13,61,64,65]. Created in BioRender. Pałucha-Poniewiera, A. (2025) https://BioRender.com/0xfrbdi (accessed on 17 November 2025).

When examining how ketamine affects the regulation of the mGlu_5_ receptor, it is important to note that this receptor can exhibit constitutive (agonist-independent) activity. Homer proteins bind directly to the carboxy-terminal intracellular domains of the mGlu_5_ receptor and regulate this activity. Research by Ango et al. [65] has shown that increased levels of Homer1a can induce constitutive activity in the mGlu_5_ receptor within neurons. As previously mentioned, activating the mGlu_5_ receptor seems to inhibit the antidepressant effects of ketamine [69]. This implies that pharmacologically blocking the mGlu_5_ receptor may enhance the antidepressant effects of ketamine. This potential benefit may explain why co-administering ketamine with mGlu_5_ receptor NAMs is effective. The proposed mechanisms of action of the mGlu_5_ antagonist in combination with ketamine or its enantiomers are comprehensively shown in Figure 2.

## 8. Conclusions

**Figure 3:**
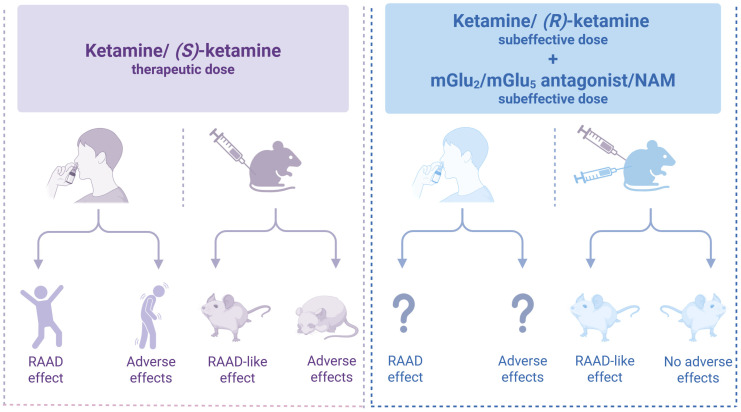
Co-administration of subthreshold doses of ketamine or (*R*)-ketamine with an mGlu2 o mGlu5 antagonist or NAM produces antidepressant-like effects [21,38,51,53,54,56,59,64,69] similar to (*S*)-ketamine [8,12,13,15,29,30,35,38,53,54] but without adverse effects [38,53,54]. Created in BioRender. Pałucha-Poniewiera, A. (2025) https://BioRender.com/0wk7zeg (accessed on 17 November 2025).

Improving the quality of therapy involves enhancing both the efficacy and safety of medications. In the case of (*S*)-ketamine, used for the treatment of TRD, the side effects play a significant role in its overall action profile. Addressing these undesirable effects poses a considerable challenge in modern psychopharmacology. One strategy to mitigate these effects is to use substances that modulate glutamatergic transmission through mGlu receptors. Preclinical research has shown that certain ligands for these receptors, including mGlu_2_ and mGlu_5_ antagonists or NAMs, resemble the cellular and molecular mechanisms of ketamine and its enantiomers. Blocking these receptors may enhance the antidepressant effects of ketamine or its safer enantiomer, (*R*)-ketamine, enabling a lower therapeutic dose while reducing the likelihood of adverse effects (Figure 3).

It is worth noting that this review expands the range of mGlu receptor ligands that enhance the effects of ketamine to include the partial NAM of the mGlu_5_ receptor. As a result, the potential for a synergistic effect with ketamine in producing antidepressant-like outcomes is not confined to mGlu_2_ receptor ligands, which have been the primary focus of research in this area for nearly a decade. Furthermore, in our analysis of the enhancement of ketamine’s action by mGlu ligands, we propose for the first time that (*R*)-ketamine, rather than (*S*)-ketamine, may be involved in this mechanism, thus opening avenues for further research.

The therapeutic approach proposed in this review could potentially improve the safety profile of ketamine and its enantiomers in the treatment of depression. However, further research, especially clinical trials, including evaluation of the antidepressant effects and safety profile of combined low doses of ketamine enantiomers with mGlu_2_ and mGlu_5_ receptor antagonists, is necessary to validate these findings. Until we obtain the results of these studies, the question marks in Figure 3 remain. Thus, the hypothesis presented in this work has not been clinically validated, which is a significant limitation. However, a review of preclinical studies showing promising results may encourage the implementation of the proposed strategy in clinical settings. Only by doing so can we determine whether the findings from studies using animal models correlate with the effects observed in patients.

## Figures and Tables

**Table 1:** Antidepressant-like effects of co-administering low, subthreshold doses of ketamine, its enantiomers, or metabolite with low, subthreshold doses of an mGlu_2_ or mGlu_5_ receptor antagonist or NAM in animal models of depression and screening tests.

Treatment	Species/Behavioral Model/Test	Effect	Reference
(*R*,*S*)-ketamine (3 mg/kg) +mGlu_2_R antagonist (LY341495) (0.3 mg/kg)	mouse/CUMS/Splash	Anti-apathetic	[38]
	mouse/CUMS/SPT	Anti-anhedonic	
	mouse/CUMS/TST	AD-like	
	mouse/CUMS/FST	No effect	
(*R*,*S*)-ketamine (3 mg/kg) +mGlu_2_R antagonist (LY341495) (0.3 mg/kg)	rat/FST	AD-like	[53]
(*R*,*S*)-ketamine (3 mg/kg) +mGlu_2_R antagonist (LY341495) (0.1 mg/kg)	rat/FST	AD-like	[56]
(*R*,*S*)-ketamine (1 mg/kg) +mGlu_2_R antagonist (LY341495) (0.1 mg/kg)	mouse/FST	AD-like	[51]
(*2R*,*6R*)-*HNK* (1 mg/kg) +mGlu_2_R antagonist (LY341495) (0.1 mg/kg)	mouse/FST	AD-like	[51]
(*R*)-ketamine (1 mg/kg) +mGlu_2_R antagonist (LY341495) (0.3 mg/kg)	mouse/TST	AD-like	[54]
	mouse/CUMS/Splash	Anti-apathetic	[54]
	mouse/CUMS/SPT	Anti-anhedonic	[54]
	mouse/CUMS/TST	AD-like	[54]
	mouse/CUMS/FST	AD-like	[59]
(*S*)-ketamine (1 mg/kg) +mGlu_2_R antagonist (LY341495) (0.3 mg/kg)	mouse/TST	AD-like	[54]
	mouse/CUMS/Splash	No effect	
	mouse/CUMS/SPT	No effect	
	mouse/CUMS/TST	No effect	
(*R*,*S*)-ketamine (1 mg/kg) +mGlu_5_R antagonist (MTEP) (1.25 mg/kg)	rat/FST	AD-like	[69]
(*R*)-ketamine (1 mg/kg) +mGlu_5_R NAM (M-5MPEP) (3 mg/kg)	mouse/TST	AD-like	[21]
	mouse/CUMS/Splash	Anti-apathetic	[64]
	mouse/CUMS/SPT	Anti-anhedonic	[64]
	mouse/CUMS/TST	AD-like	[64]
(*S*)-ketamine (1 mg/kg) +mGlu_5_R NAM (M-5MPEP) (3 mg/kg)	mouse/TST	No effect	[21]

## Data Availability

Not applicable.

## Abbreviations

The following abbreviations are used in this manuscript:

AMPA	alpha-amino-3-hydroxy-5-methyl-4-isoxazolepropionic acid
BDNF	brain-derived neurotrophic factor
BOLD	blood oxygenation level-dependent
CUMS	chronic unpredictable mild stress
eEF2	eukaryotic elongation factor 2
FST	forced swim test
GABA	gamma-aminobutyric acid
HNK	hydroxynorketamine
LTP	long-term potentiation
mGlu receptor	metabotropic glutamate receptor
M-5MPEP	2-(2-(3-methoxyphenyl)ethynyl)-5-methylpyridine
mPFC	medial prefrontal cortex
mTOR	mammalian target of rapamycin
NAM	negative allosteric modulator
NMDA	*N*-methyl-D-aspartic acid
NOR	novel object recognition
PAM	positive allosteric modulator
PCP	phencyclidine
PET	positron emission tomography
PFC	prefrontal cortex
phMRI	pharmacologic magnetic resonance imaging
PSD-95	postsynaptic density 95
qEEG	quantitative electroencephalography
RAAD	rapid-acting antidepressant drug
SDS	social defeat stress
SSRI	selective serotonin reuptake inhibitor
TRD	treatment-resistant depression
TrkB	tropomyosin receptor kinase B
TST	tail suspension test
